# The Contribution of Chemistry to the Detection and Enumeration of *Legionella pneumophila* in Environmental Water Samples: Experience With the MICA Method

**DOI:** 10.1002/mbo3.70295

**Published:** 2026-06-12

**Authors:** Savina Ditommaso, Jacopo Garlasco, Carla Streva, Gabriele Memoli, Carla M. Zotti, Fabrizio Bert, Monica Giacomuzzi

**Affiliations:** ^1^ Department of Public Health and Pediatrics University of Turin Turin Italy; ^2^ Infectious Diseases Unit, Department of Diagnostics and Public Health University of Verona Verona Italy

## Abstract

*Legionella* is widespread in natural aquatic habitats and can contaminate man‐made water systems. Due to public‐health risks, measuring microbial load in water samples is essential. This study compared a new lipopolysaccharide bioprobe method (Microcolony Counter Analysis—MICA), which detects and counts *Legionella pneumophila* in 2 days, with the standard culture method (ISO 11731:2017), which may take up to 10 days. Our results on 108 water samples showed 82.4% agreement. Fifteen were ISO+/MICA− and four ISO−/MICA+; MICA sensitivity was 67.4%. Analysis of culture‐media factors (*Legionella* inhibition by contaminating flora; culture on Glycine Vancomycin Polymyxin Cycloheximide agar (GVPC) and MICA factors (possible killing/growth inhibition due to acid treatment) showed that (a) ISO counts tended to be higher than MICA, with little difference whether GVPC results or the maximum yield between Buffered Charcoal Yeast Extract agar (BCYE) and GVPC were used. (b) Acid‐treated MICA samples tended to yield higher counts than untreated ones, especially at high MICA counts. Considering what has been disclosed, a short 48‐h incubation may reduce MICA recovery for some wild *L*. *pneumophila* strains, affecting click‐based detection. With refinement, MICA could be a practical, user‐friendly diagnostic tool, simpler sample prep, no large‐volume filtration, no colony isolation or extra confirmation and provides confirmed results in 2 days versus ≥ 10 days for ISO culture.

## Graphical abstract

*Legionella pneumophila* is the principal species responsible for Legionnaires' disease, and measuring microbial load is fundamental to the analysis of environmental water samples. This study compared a new lipopolysaccharide bioprobe method (Microcolony Counter Analysis), which detects and counts *L. pneumophila* in 2 days, with the standard culture method (ISO 11731:2017), which may take up to 10 days.

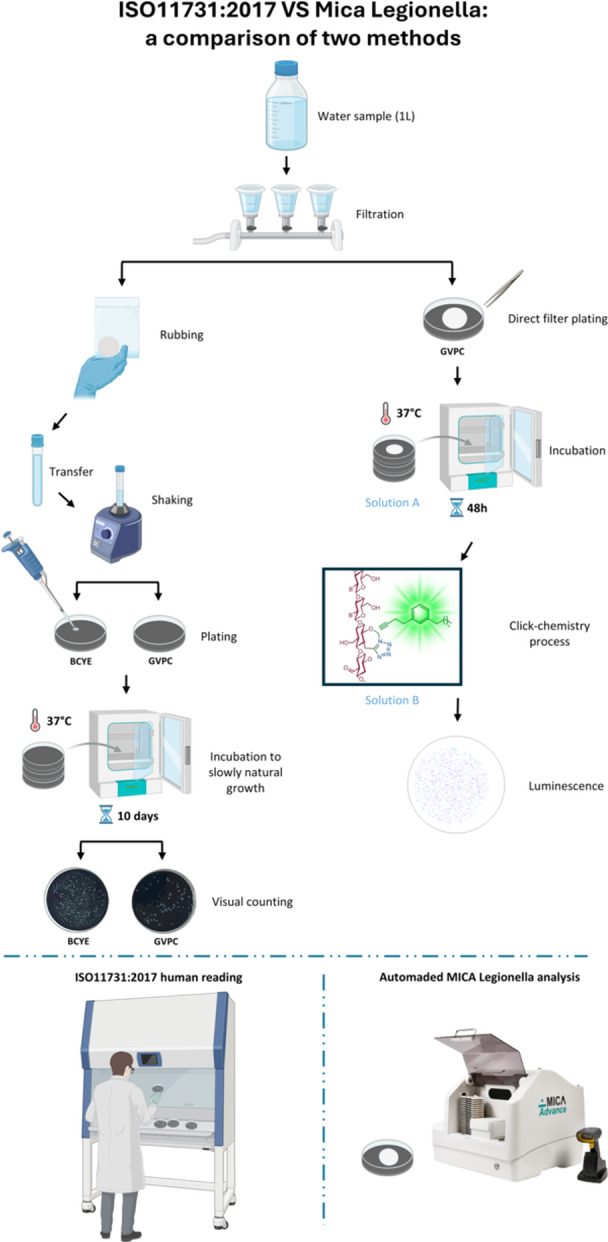

## 1 Introduction

*Legionella* is widely present in aquatic environments. From its natural reservoir (e.g., lakes, rivers, and thermal springs), it can enter and colonize artificial water systems (e.g., distribution networks, storage tanks, and cooling towers), and subsequently reach humans (Wadowsky et al. 1982; Fliermans 1996; van Heijnsbergen et al. 2015). Although studies have not shown a direct correlation between *Legionella* concentrations and the risk of legionellosis (Best et al. 1983; Kool et al. 1999; Stout et al. 2007), the consensus in environmental management is that reducing *Legionella pneumophila* concentrations below < 1000 CFU/L is necessary to manage public‐health risks or susceptible population settings (Hamilton et al. 2019; European Union 2020; Kanarek et al. 2022).

The risk of legionellosis is not solely based on concentration, but rather a combination of factors. Key findings on the relationship between concentration and risk include (i) aerosol generation: high concentration in water only leads to high risk if that water is aerosolized (showers, cooling towers, and fountains); (ii) temperature (20°C and 45°C are optimal for growth, leading to higher risks); (iii) water stagnation (poorly circulating water allows *Legionella* populations to increase, increasing the risk); (iv) chlorine levels (chlorine levels < 0.2 mg/L are associated with higher *Legionella* colonization); host vulnerability (while high concentrations are riskier for everyone, lower concentrations can still pose a risk to highly immunocompromised individuals); (v) species variation. It is widely demonstrated that the most pathogenic species is *L. pneumophila* and that among *L. pneumophila*, the principal species responsible for Legionnaires' disease is *L. pneumophila* serogroup 1, which, in 2021, accounted for 89% of reported cases in Europe and 96.9% in the United States (European Centre for Disease Prevention and Control 2023; Centers for Disease Control and Prevention 2025).

Therefore, measuring microbial load of *Legionella* and its serotyping on environmental water samples is necessary to manage public‐health risks. When choosing culture methods, maximum sensitivity for detecting *Legionella* is preferable because of the public‐health implications of the results.

The most commonly used culture technique for environmental surveillance of *Legionella* is the standard method according to ISO 11731:2017 (International Standard Organisation 2017), which allows isolation of *Legionella* organisms and estimation of their numbers in environmental samples after sample concentration.

Our laboratory is involved in environmental investigation for *Legionella* testing for numerous hospitals and facilities to verify the effectiveness of sanitation and disinfection procedures.

We developed and validated ISO 11731 protocols (Ditommaso et al. 2011), and we are aware that several factors in the ISO method can hinder accurate *Legionella* detection and quantification (membrane type, heat or acid treatment, medium, and methods used to detach cells from the membrane).

Moreover, the culture method is suitable only for laboratories with established expertise in distinguishing *Legionella* from the myriad other bacteria found in water.

Alternative cultural and molecular methods that attempt to overcome limitations of the standard method (i.e., long turnaround times: ISO method may require as long as 10 days to detect *Legionella* in a water supply) are outlined (Ditommaso et al. 2010, 2014; Lee et al. 2015; Rech et al. 2018; Fisher et al. 2020; Fricke et al. 2020; Nácher‐Vázquez et al. 2022); however, they still have limitations.

Among the alternative culture methods in literature, experiences with the ScanVIT‐*Legionella* and Legiolert methods are reported. A major limitation of the ScanVIT‐*Legionella* method is that it does not allow for the recovery of *Legionella* bacteria from the filter for further cultivation, typing, or molecular analysis. This is a crucial drawback for epidemiological investigations needing to link environmental sources to clinical cases (Ditommaso et al. 2010; Gruas et al. 2013). On the other hand, for the Legiolert method, some studies have demonstrated a false positivity result (rate between 0% and 4%) due to waterborne bacteria, such as *Pseudomonas* spp., *Proteus* spp., and *Stenotrophomonas* spp. (LeChevallier et al. 1980; Hirsh et al. 2021; Donohue et al. 2023). Molecular assays that target *Legionella* DNA are highly fast, sensitive, and specific, can differentiate species and serogroups, and can detect viable‐but‐nonculturable organisms. However, they are not ideal for reliable quantification in water samples because they cannot consistently distinguish DNA from live cells versus dead cells (Koide et al. 1993; M. N. Bates et al. 2000). Applying the quantitative PCR (qPCR) combined with propidium monoazide (PMA) treatment (Nocker et al. 2006; Contreras et al. 2011; Slimani et al. 2012; Ditommaso et al. 2014) it is possible to obtain a reduction in qPCR signal from dead cells but there is a dissimilarity in the ability of PMA to suppress the PCR signal in samples with different amounts of bacteria: the effective elimination of detection signals by PMA depended on the concentration of genomic unit (GU) and increasing amounts of cells resulted in higher values of reduction. Therefore, even the use of PMA does not guarantee the complete absence of signals deriving from dead cells. As of now, it is difficult to compare qPCR results with those obtained by culture, because qPCR results are reported in GUs while culture results are reported as colony‐forming units (CFUs), which is the metric used in most technical guidelines for the prevention, control, and investigation of infections caused by *Legionella* species (Joseph et al. 2011; Ministero della Salute 2015; Centers for Disease Control and Prevention 2004).

A recent diagnostic test for environmental analysis (Passot et al. 2024) is the Microcolony Counter Analysis (MICA) (Diamidex, Marseille, France), which uses a chemical reaction known as “click chemistry” with a lipopolysaccharide bioprobe (Mas Pons et al. 2014). By adding a specific modified sugar (pLeg‐N3) to the growth medium, the growing *L. pneumophila* cells internalize this sugar and incorporate it into their cell wall. The main advantage of the MICA method is significant time reduction (48 h): thanks to “click chemistry” the MICA method is enabled to detect and quantify all serogroups of cultivable *L. pneumophila* with automatic enumeration within 2 days and reports results directly in CFUs, providing direct continuity with existing risk management guidelines and action levels. Moreover, the method significantly reduces handling time per sample allowing for higher reproductivity and eliminates the possibility of nonrecognition legionella colonies by microbiologists. Indeed, the most challenging task in *Legionella* detection is represented by the ability to recognize all colonies belonging to the *Legionella* genus.

This study aims to comparatively assess the performance of MICA against ISO 11731:2017 for quantifying *L. pneumophila* in environmental water samples under real environmental conditions.

## 2 Methods

Hot‐water samples were collected during routine investigation from in‐building water distribution systems of nine healthcare facilities, four residential buildings, and one medical clinic, all located in the Piedmont region of Italy. Facilities were chosen to be representative of locations that host high‐risk groups (healthcare facilities) or that exhibit conditions conducive to *Legionella* growth (residential buildings). Sampling was performed after conducting an environmental risk assessment of the building water systems to identify potentially hazardous conditions. We developed the environmental sampling plans, and hot‐water systems were considered for environmental water sampling because they were the primary source of Legionnaires' disease (temperatures between 20°C and 50°C are optimal for *Legionella* growth).

Each sample was collected in sterile 1‐L plastic bottles. Sodium thiosulfate solution (20 mg/L) was added to the samples to neutralize free chlorine in treated water supplies. The samples were transported to the laboratory at room temperature and processed on the day of collection using both the MICA and ISO 11731:2017 methods. Both analyses were performed on two aliquots from the same sample.

### 2.1 Culture According to ISO 11731:2017 Method

Analyses to quantify *Legionella* spp. were performed according to ISO 11731:2017 (Ditommaso et al. 2011, 2022).

Briefly, water samples were concentrated by filtration through 0.22‐μm polyethersulfone filters (Millipore, Billerica, MA, USA). After filtration, each filter was aseptically placed in a bottom corner of a stomacher bag containing Page solution (pH 6.8) and rubbed for 1 min to detach bacteria. A 0.2‐mL aliquot of the concentrated sample was plated onto BCYE and GVPC agar plates (Thermo Fisher Scientific, Germany), which were then incubated at 36°C for 10 days.

The plates were checked at days 2, 3, 5, and at the end of the incubation period. In case of high concentration of interfering microorganisms on day 2, the concentrated samples, stored at 5°C ± 3°C, were plated after dilution, acid treatment, and thermal treatment (this step allowed us to identify samples where overgrowth had occurred according to the suggestion of ISO 11731).

Suspected *Legionella* colonies are examined under an ultraviolet lamp to identify autofluorescent colonies: brilliant white (e.g., *Legionella anisa*, *L. bozemanii*, and *L. dumoffii*), red (e.g., *L. erythra* and *L. rubrilucens*), or dull green often with a yellow tinge (e.g., *L. pneumophila*). The fluorescence color can aid in distinguishing different *Legionella* species.

Presumptive *Legionella* colonies were confirmed by subculturing them on blood (Thermo Fisher Scientific, Germany) and BCYE agar plates. Colonies grown only on BCYE agar were identified by means of an agglutination test (*Legionella* latex test, Thermo Fisher Scientific, UK).

Agglutination‐negative isolates underwent further analysis by a laboratory‐developed PCR assay targeting *Legionella* spp. 16S ribosomal RNA gene, following the protocol of Miyamoto et al. (1997). The plate yielding the greatest number of confirmed colonies was used to calculate the *Legionella* spp. concentration in the original sample. Results are reported as CFU/L. Given our concentration procedure, the method detection limit was 50 CFU/L.

### 2.2 Culture According to the MICA *Legionella* Method

**Figure 1:**
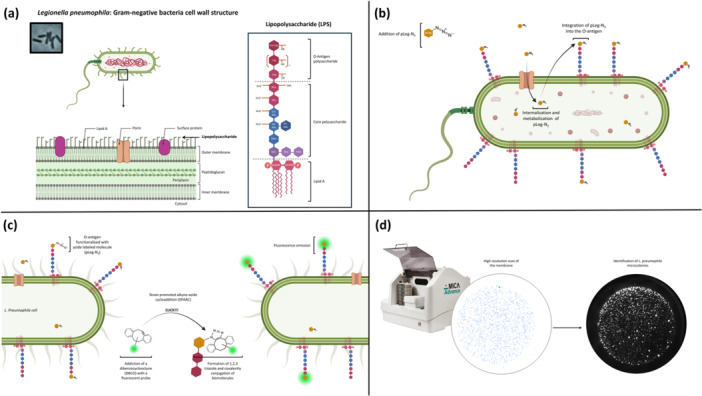
Specific tagging of *Legionella pneumophila* automatically detected at a microcolony stage by solid‐phase cytometry using the MICA microcolony counter. (a) *L. pneumophila*: Gram‐negative bacteria cell wall structure, (b) treatment with solution A (Diamidex‐patented molecules), (c) tagging with solution B (bind specifically by click chemistry onto the bio‐orthogonal azido group, and (d) *L. pneumophila enumeration by* MICA microcolony counter. MICA, Microcolony Counter Analysis.

Briefly, water samples were concentrated by filtration using MICA filters (polyvinylidene fluoride). After filtration, the filters were treated with pH 2 solution at room temperature for 5 min; some samples were collected in duplicate and analyzed with and without acid treatment. Filters were rinsed with sterile water, placed on GVPC plates layered with MICA solution A (Figure 1b) and incubated at 37°C for 48 h. This step allows microcolonies of *L. pneumophila* to form and be labeled by Diamidex's patented molecule. After incubation, each membrane was overlaid with tagging solution B (Figure 1c). This step (at 37°C for 15 min) tags the microcolonies with a fluorescent molecule, via a click‐chemistry reaction that links the fluorophore to the Diamidex‐patented molecule bound to the bacteria. After the 15‐min incubation, the membrane is washed for 15 min to remove excess fluorescent dye and then read with the Microcolony Counter equipment (Figure 1d).

The MICA Fluorescence Counter enables counting objects (such as microcolonies) on a membrane. Its optical system takes very high‐resolution snapshots of each membrane, which are then analyzed by the MICA Fluorescence counter's software to determine the precise number of objects present on the membrane. Fluorescent labeling permits automatic detection of CFUs at the microcolony stage via solid‐phase cytometry: the MICA microcolony counter performs a high‐resolution scan of the membrane. The MICA *Legionella* artificial intelligence (AI) analyzer then evaluates multiple features to specifically recognize *L. pneumophila* microcolonies (as low as 2 CFU/test portion) and reports the result as the concentration of *L. pneumophila* in the original sample. For better reproducibility, the MICA software provided with the microcolony counter provides a step‐by‐step protocol guide, including control of the incubation times and reagent traceability. Its built‐in guidance and automated analysis make the MICA *Legionella* workflow accessible to users of varying experience. The AI module within the MICA software automatically detects *L. pneumophila* microcolonies on the membrane by performing a multiparametric image analysis and directly reports the concentration of *L. pneumophila* in the water sample. On the basis of the initial filtrate volume, results are reported as CFU/L of *L. pneumophila*. No human interpretation or calculations are required, which minimizes user‐to‐user variability and improves reproducibility. Results are saved within the MICA software and can be retrieved at any time or exported as comma‐separated values files or portable ducument format analysis reports. Traceability logs for each analysis are also available.

### 2.3 Data Analysis

Data were collected and organized using Microsoft Access and Excel 2016 (Microsoft Corporation, Redmond, WA, USA). Descriptive analyses were conducted and reported in terms of absolute frequencies and percentages (for categorical variables), and geometric means were provided as a synthetic measure for counts. Agreement between culture and MICA methods, in terms of positivity/negativity, was determined by comparing the results yielded by the two methods on two‐by‐two contingency tables. ISO culture was taken as the reference method.

The comparison between ISO and MICA methods, as well as between different techniques within each method—that is, nonselective/selective culture media for ISO (BCYE vs. GVPC) or the presence/absence of acid treatment for MICA—was first performed via Wilcoxon's signed‐rank test; second, counts were represented on logarithmic plots, where trend lines derived preferentially by locally estimated scatterplot smoothing (Cleveland 1979), and alternatively by generalized additive models (R package *mgcv*, Wood 2011) where the previous method could not ensure sufficient model robustness, were plotted to allow a visual evaluation of the possible nonlinear association between the respective measures that were compared.

Additionally, the difference between decimal logarithms of ISO and MICA counts for corresponding samples was evaluated through a linear mixed‐effects model (R package *lme4*, D. Bates et al. 2015), considering the random effect of the heterogeneity between samples; this difference was also assessed after adjusting for the presence or absence of acid treatment in the MICA method. This computation was performed first considering the result on selective medium only, and second considering the combined yield from nonselective and selective media, as an ISO result. All analytic computation and plotting were carried out using the statistical software R version 4.5.2 (R Foundation for Statistical Computing, Vienna, Austria) (R Development Core Team 2019). The *ggbeeswarm* package was used to arrange points in the scatterplots, so as to avoid overplotting and to reflect the density of data for high‐density values (Clarke et al. 2023).

## 3 Results

A total of 108 water samples were analyzed between May 2024 and January 2025. Samples were predominantly from hot‐water systems (83), and a smaller number of samples were from cooling towers and evaporative condensers (4) and cold water (21).

By standard method, *Legionella* spp. was isolated from 54 (50.0%), and the colony counts ranged from 50 to 1.6 × 10^5^ CFU/L (geometric mean 1.3 × 10^3^ CFU/L). *L. pneumophila* was present in 24 samples (22.2%) without the presence of any other species, whereas 22 samples (20.4%) were positive for both *L. pneumophila* and *L*. non‐*pneumophila* species, and eight (7.4%) were positive for *L*. non*‐pneumophila* species only. In all 54 positive water samples, *Legionella* grew with associated flora. Nine samples (8.3%) had high concentrations of additional microbial flora on both the selective (GVPC) and nonselective medium (BCYE) (unreadable plates), and for three of these samples, the *L. pneumophila* (two samples) and *L*. non‐*pneumophila* species (one sample) count was performed using plates inoculated with a portion of the acidic and heat‐treated sample (Table 1).

**Table 1:** *Legionella* enumeration (CFU/L) by ISO and by MICA methods.

Sample	Matrix	Sample ID number	BCYE plate result	GVPC plate result	Combined ISO result^c^ [max(GVPC, BCYE)]	*Legionella pneumophila* serogroups	MICA result
1	Hot water	26028	Overgrowth	1750	1750	*L.p*. sg 6	≤ 8
2	Hot water	26029	Overgrowth	400	400	*L.p*. sg 6	104
3	Cold water	26032	Overgrowth	600	600	*L.p*. sg 1	215
4	Hot water	26033	Overgrowth	2600	2600	*L.p*. sg 1	1808
5	Cold water	26042	50	100	100	*L.p*. sg 1	9
6	Cold water	26043	550	500	550	*L.p*. sg 1	< 1
7	Hot water	26053	60,000	10,000	60,000	*L.p*. sg 2	> 40,000
8	Hot water	26054	1450	1100	1450	*L.p*. sg 2	4782
9	Hot water	26072	450	450	450	*L.p*. sg 1	< 1
10	Hot water	26073	350	50	350	*L.p*. sg 1	< 1
11	Hot water	26145	50	< 50	50	*L.p*. sg 6	< 1
12	Hot water	26149	50	50	50	*L.p*. sg 6	< 1
13	Hot water	26157	600	100	600	*L.p*. sg 6	98
14	Hot water	26223	7150	7750	7750	*L.p*. sg 3	12,532
15	Hot water	26224	1450	1150	1450	*L.p*. sg 3	1107
16	Hot water	26225	50,000	60,000	60,000	*L.p*. sg 3	> 40,000
17	Hot water	26231	100	50	100	*L.p*. sg 2‐14^b^	< 1
18	Cold water	26307	9650	6800	9650	*L.p*. sg 1	59,211
19	Water^d^	26354	Overgrowth	160,000	160,000	*L.p*. sg 2‐14^b^	> 80,000
20	Cold water	26459	< 50	100	100	*L.p*. sg 2	403
21	Cold water	26460	300	200	300	*L.p*. sg 1	554
22	Hot water	26461	< 50	1050	1050	*L.p*. sg 1	438
23	Cold water	26482	100	100	100	*L.p*. sg 1	≤ 4
24	Cold water	26484	250	250	250	*L.p*. sg 1	59
25	Cold water	26485	Overgrowth	850	850	*L.p*. sg 1	956
26	Hot water	26522	Overgrowth	33,000	33,000	*L.p*. sg 1	≤ 4
27	Hot water	26523	Overgrowth	51,000	51,000	*L.p*. sg 2	≤ 4
28	Hot water	26648	Overgrowth	19,000	19,000	*L.p*. sg 2	> 80,000
29	Hot water	26669	Overgrowth	4200	4200	*L.p*. sg 3	4902
30	Hot water	26670	Overgrowth	1650	1650	*L.p*. sg 1	3001
31	Hot water	26672	< 50	50	50	*L.p*. sg 1	29
32	Hot water	26673	Overgrowth	350	350	*L.p*. sg 1	452
33	Hot water	26674	Overgrowth	150	150	*L.p*. sg 5	< 1
34	Hot water	26675	2600	2200	2600	*L.p*. sg 5	4917
35	Hot water	26676	Overgrowth	Overgrowth	1800^a^	*L.p*. sg 1	≤ 4
36	Hot water	26677	Overgrowth	Overgrowth	12,200^a^	*L.p*. sg 1	765
37	Hot water	26678	Overgrowth	350	350	*L.p*. sg 3	280
38	Cold water	26882	200	150	200	*L.p*. sg 1	≤ 4
39	Cold water	26883	Overgrowth	1000	1000	*L.p*. sg 1, *L.p*. sg3	360
40	Hot water	26904	Overgrowth	3550	3550	*L.p*. sg 2	6229
41	Hot water	26910	2600	1900	2600	*L.p*. sg 2	960
42	Hot water	27027	200	50	200	*L.p*. sg 6	13
43	Hot water	27028	50	< 50	50	*L.p*. sg 1	7
44	Cold water	27033	350	50	350	*L.p*. sg 1	164
45	Cold water	27297	Overgrowth	200	200	*L.p*. sg 1	≤ 4
46	Cold water	27298	50	150	150	*L.p*. sg 1	≤ 4
47	Hot water	26026	Overgrowth	< 50	< 50	—	4
48	Water^d^	26169	Overgrowth	Overgrowth	< 50	—	40,000
49	Hot water	26232	< 50	< 50	< 50	—	76
50	Cold water	26353	< 50	< 50	< 50	—	32

Abbreviations: CFU, colony‐forming unit; ISO, International Standard Organisation; MICA, Microcolony Counter Analysis.

^a^ *Legionella* enumeration was performed using the plates inoculated after acid treatment combined with heat treatment.

^b^ *Legionella* strains are positive by polyvalent agglutination serum 2–14 with cross‐agglutination by individual sera of serogroups from 2 to 14.

^c^ According to ISO methods, to estimate the number of colonies forming unit of *Legionella* in the original water samples we selected the plates showing the maximum number of confirmed colonies per water volume.

^d^ Water: Samples collected from cooling towers and evaporative condensers.

By the MICA method, *L. pneumophila* was detected in 35 (32.4%). Three samples that tested negative with the culture method but positive with the MICA method had a *Legionella* count 6, 32, and 76 CFU/L, which were below and around the detection limit of the culture method, and one sample had a *Legionella* count 4.0 × 10^4^ CFU/L (this sample was taken from a cooling tower). The 15 samples that tested negative with the MICA but positive with the culture method had a *Legionella* concentration between 50 and 5.1 × 10^4^ CFU/L; only one of these showed overgrowth on the GVPC medium, and the *Legionella* count (1.8 × 10^3^ CFU/L) was performed using the plates inoculated after acid treatment combined with heat treatment (Table 1). By the ISO method, the colony counts of *L. pneumophila* ranged from 50 to 1.6 × 10^5^ CFU/L (geometric mean 9.6 × 10^3^ CFU/L); by the MICA method, *L. pneumophila* the colony counts ranged from 4 to 8.0 × 10^4^ CFU/L (geometric mean 7.3 × 10^2^ CFU/L). The most frequently isolated serogroups were the *L. pneumophila* serogroup 1 (51.1%). Details of positive samples by serogroups are shown in Table 1.

**Figure 2:**
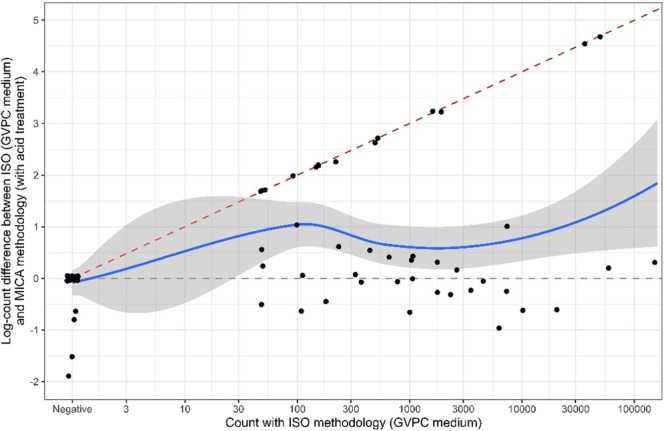
Difference between ISO (GVPC) and MICA counts in relation to the actual ISO value. The blue curve represents the trend of the ISO–MICA log difference across the ISO values, while the gray ribbon represents the 95% confidence interval for this trend. The horizontal dashed gray line represents the nondifference line between ISO and MICA, while the oblique dashed red line represents samples that turned positive with the ISO method only (i.e., for which log MICA was zero and, therefore, the difference between log ISO and log MICA was equal to log ISO). ISO, International Standard Organisation; MICA, Microcolony Counter Analysis.

The scatter plot in Figure 2 shows that the concentration of *L. pneumophila* highlighted in the sample through the ISO method does not influence the difference between the two techniques. For different values of the ISO count, the trend of the ISO–MICA difference was quite stable (only slightly increasing), and also the variability of this difference—as expressed by the width of the confidence interval for this trend—was quite constant regardless of the actual count.

The MICA method had a sensitivity of 67.4%; agreement between the two methods was 82.4%. Calculation of the coefficient of Cohen's kappa showed good concordance between the two methods (*κ* = 0.629) (Table 2).

**Table 2:** Comparison of *Legionella pneumophila* recovery obtained with different culture methods.

	ISO 11731:2017		
	Positive (*n*)	Negative (*n*)	Total (*n*)
*MICA*			
Positive (*n*)	31	4^c^	35
Negative (*n*)	15	58	73
Total (*n*)	46^b^	62^a^	108

*Note:* Agreement = 82.4%; *κ* = 0.629; Sensitivity = 67.4%.

Abbreviations: ISO, International Standard Organisation; MICA, Microcolony Counter Analysis.

ISO method:

a Six samples with overgrowth of background flora.

b Two samples with overgrowth of background flora.

c One sample with overgrowth of background flora.

**Figure 3:**
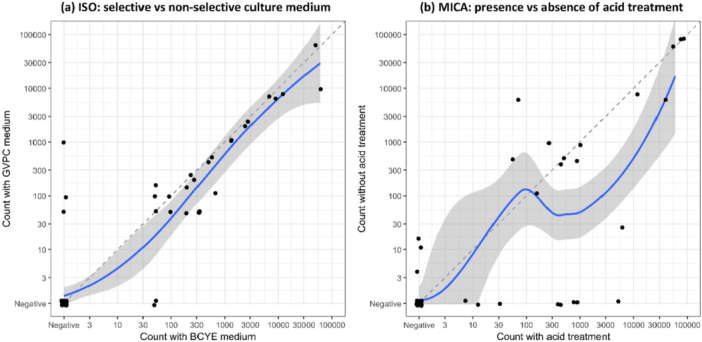
Observed counts in the presence of different methods for the same technique. (a) ISO: selective (GVPC) versus nonselective medium (BCYE); (b) MICA: presence versus absence of prior acid treatment. The dashed line indicates no difference between the methods, the blue line shows the nonlinear association between variables (and its 95% confidence interval). ISO, International Standard Organisation; MICA, Microcolony Counter Analysis.

For the 22 samples in which *Legionella* was identified on both the selective (GVPC) and nonselective medium (BCYE), a significant difference emerged between the two media, suggesting greater counts for the nonselective medium (Wilcoxon test, *p* = 0.011); however, the average difference between counts did not exceed 0.2 log (the geometric mean of the ISO counts was 7.7 × 10^2^ and 5.0 × 10^2^ on BCYE and GVPC, respectively); all positive results yielded by the ISO test are reported in Table 1. Moreover, considering all samples, BCYE often showed similar yields compared with GVPC counts (Figure 3a).

Sixty‐four samples were analyzed by MICA with and without acid treatment, and the detection of *L. pneumophila* was 34.4% (22/64) and 26.6% (17/64), respectively. Among these 64 samples, the 14 (21.9%) that yielded a positive result both with and without acid treatment failed to show a difference between the two techniques (*p* = 0.610). However, considering all specimens, samples undergoing prior acid treatment tended to show greater yields compared with those without treatment, especially in the event of high MICA counts (Figure 3b).

**Figure 4:**
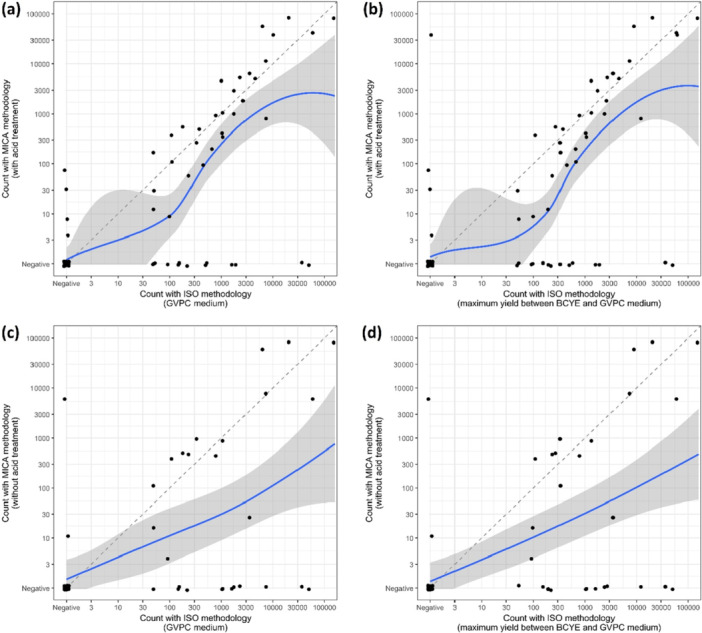
Comparison between ISO and MICA techniques, according to the respective methods: (a) ISO selective medium versus MICA with acid, (b) ISO combined result (BCYE + GVPC) versus MICA with acid, (c) ISO selective medium versus MICA without acid, and (d) ISO combined result (BCYE + GVPC) versus MICA without acid. The dashed line indicates no difference between the methods, and the blue line shows the nonlinear association between variables (and its 95% confidence interval). ISO, International Standard Organisation; MICA, Microcolony Counter Analysis.

Considering only the 31 samples that tested positive with both ISO and MICA methods, the *Legionella* concentration detected by the culture method was not significantly different from that detected with the MICA method (Wilcoxon test, *p* = 0.645). However, taking all the samples into account, a trend was observed, suggesting greater counts with the ISO method, apparently also driven by the presence of 15 samples which gave a positive result with the ISO method only (Figure 4). This trend was observed regardless of the culture medium, or of the presence of acid treatment before MICA evaluation, even though with a different extent and uncertainty accordingly (Figure 4a–d).

The models considering heterogeneity between samples are in agreement with the observed trends, with a predicted difference in counts in favor of the ISO method (Table 3). This difference retained significance (*p* < 0.001) considering either the crude yield on the selective medium (Table 3a) or the maximum between selective and nonselective media (Table 3b) as ISO result, with a difference around 0.4–0.6 log both in the unadjusted and in the adjusted models. The predicted difference between ISO and MICA appeared to be slightly for MICA observations with prior acid treatment (*p* = 0.043).

**Table 3:** Predicted difference between log‐counts obtained with ISO and MICA techniques. Unadjusted and adjusted models are provided, considering heterogeneity between samples and settings, as ISO result, either selective medium counts (a) or the maximum between selective and nonselective medium counts (b).

Univariate analysis			
**Variable**	**Estimate**	**95% CI**	***p* value**
**(a) ISO: Selective medium only**			
Difference [log(ISO) − log(MICA)]	0.398	0.176; 0.620	< 0.001
**Multivariable analysis (after adjustment for MICA acid treatment)**			
Difference [log(ISO) − log(MICA)]	0.569	0.252; 0.886	< 0.001
MICA treatment (ref = no acid)			
Acid treatment	−0.257	−0.505; −0.009	0.043
**(b) ISO: Maximum count between selective and nonselective medium**			
Difference [log(ISO) − log(MICA)]	0.420	0.189; 0.652	< 0.001
**Multivariable analysis (after adjustment for MICA acid treatment)**			
Difference [log(ISO) − log(MICA)]	0.605	0.284; 0.927	< 0.001
MICA treatment (ref = no acid)			
Acid treatment	−0.250	−0.490; −0.009	0.043

Abbreviations: CI, confidence interval; ISO, International Standard Organisation; MICA, Microcolony Counter Analysis.

## 4 Discussion

Current detection methods for *Legionella* in environmental and clinical samples are time‐consuming (10–14 days for results), labor‐intensive (Lucas et al. 2011; Public Health England 2015; International Standard Organisation 2017; Centers for Disease Control and Prevention 2022) and require substantial expertise in recognizing *Legionella* colonies. *Legionella* counts can be underestimated because viable‐but‐nonculturable cells or bacteria residing inside amoebae may not be detected (Kirschner 2016; Boamah et al. 2017; Nisar et al. 2023).

The data presented here describe a field investigation that compared the ISO 11731 method with an alternative method, MICA *Legionella*, a new method for identifying and enumerating *L. pneumophila* in environmental water samples. The data obtained show that the two methods were comparable (agreement = 82.4%). However, 15 water samples were positive by the ISO method and negative by MICA, while only four samples were negative by ISO and positive by MICA. Among these four samples, the one collected from the cooling tower (Sample No. 48, Table 1) contained 4 × 10^4^ CFU/L of *L. pneumophila* detected by MICA. On complex matrices such as cooling tower waters, MICA's rapid 48‐h timeframe prevents interfering background flora from overgrowing the plates, yielding more reliable results than traditional culture.

Overall, the sensitivity of the MICA test was 67.4%.

This result disagrees with findings from other authors: Passot et al. (2023) compared the performance and robustness of MICA *Legionella* for the enumeration of culturable *L. pneumophila* with the reference method ISO 11731:2017 and with AFNOR standard methods NF T90‐431 (Agence Française de Normalisation 2003; Passot et al. 2024).

In these previous studies, they concluded that enumeration of *L. pneumophila* by MICA in cooling tower waters showed better sensitivity than ISO 11731:2017 (Passot et al. 2023: sensitivity = 94%), and that the MICA method was statistically equivalent to the French reference method for recovering *Legionella* in domestic hot water (Passot 2024: MICA sensitivity = 91.4%). Our results do not confirm the findings of previous investigations: it should be noted that those results were obtained using different plate‐culture procedures (sample volume plated and culture media) analyzed different types of samples (spiked samples, samples with high *Legionella* load) (Passot et al. 2023) or used different methods for results calculation/interpretation (see supporting file of Passot et al. 2024).

It should be emphasized that the traditional “gold standard” for detecting *Legionella* in water samples is a complex plate‐culture method involving numerous steps to obtain confirmed results. International organizations (Agence Française de Normalisation 2003; Public Health England 2015; International Standard Organisation 2017; Centers for Disease Control and Prevention 2022) have published procedures but the application of these techniques can still require considerable prior experience and expertise from the laboratorian. Each sample preparation step represents opportunities for bacterial loss, and when combined with methodological variability (sample volume filtered, media supplier, acid treatment, and filter type), the presence of competing bacteria (which complicates distinguishing *Legionella* colony morphology from the autochthonous microbiota), and subjective decisions during plate reading, this results in significant cumulative measurement uncertainty. With so many sources of variability, it is difficult to generate sufficiently reproducible intra‐ and interlaboratory results over time. In April 2024, European Centre for Disease Prevention and Control (ECDC) published a report (External Quality Assessment Schemes to Support European Surveillance of Legionnaires' Disease in EU/EEA Countries 2022–2023 2024) related to an external quality assessment schemes (EQA) organized to monitoring the accuracy of *Legionella* testing and results reported by individual laboratories in EU/EEA countries. The EQA scheme samples (10 representative samples of environmental material) were sent to each of the 24 participating laboratories: the overall isolation performance for culture was very good (88.3% averaged across all 10 samples). Across all 24 laboratories, one laboratory reported an incorrect isolation result six times, one laboratory four times, seven laboratories twice, and four laboratories once. It should be noted that this 88.3% performance was achieved by selected laboratories involved in surveillance and management of *Legionella*‐related public‐health incidents in their countries. Protocol implementation is very laboratory dependent. In our laboratory, we have analyzed environmental water samples for over 20 years (about 1500 samples/year) and consistently participate in FEPTU quality controls, always achieving high scores. Indeed, we generated performance data similar (Ditommaso et al. 2023) to those obtained during the intralaboratory trial in the primary characterization data of ISO 11731, which took place in the Netherlands (Annex H, Table H.1). Therefore, we can reasonably conclude that our sensitivity is consistent with the test's potential.

To explain the causes of this difference in sensitivity, we analyzed variables related to culture media (inhibition of *Legionella*, besides contaminating flora, on GVPC selective medium) and MICA methodology (possible *Legionella* kill or growth inhibition due to the acid treatment). The result provided by the analysis showed that: (a) counts yielded by the ISO culture tend to be higher than those obtained with the MICA method, with negligible differences according to whether the crude GVPC results or the maximum yield between BCYE and GVPC is used (as evident, e.g., by comparing Figure 4a with Figure 4b or Figure 4c with Figure 4d); this stems from the general similarity between results provided by BCYE and GVPC methods (Figure 3a). However, the nonselective medium, used as a complement to the counts obtained on GVPC, detect greater counts in 30% of the samples and thus BCYE was useful to obtain values closer to the actual amount of *Legionella*, thereby enhancing diagnostic accuracy as widely demonstrated in previous studies (Ditommaso et al. 2011, 2022; Jiménez Mayordomo et al. 2024); (b) MICA samples undergoing prior acid treatment tended to show greater yields compared with those without treatment, especially in the event of high MICA counts (Figure 3b). Moreover, the lack of acid treatment was shown to sharpen the yield difference between ISO and MICA, both in trend estimates (as appears, e.g., by comparing Figure 4a with Figure 4c or Figure 4b with Figure 4d) and in the models (Table 3), suggesting that acid treatment allows greater accuracy in detecting *Legionella* when compared with the current ISO standards. Thus, considering what has been disclosed, one explanation for the low recovery of *L. pneumophila* with the MICA method could be the short incubation time (48 h) for some wild strains of *L. pneumophila*, which may affect cell detection by click‐based technologies.

Regarding the target of MICA test, we can underline two disadvantages: (a) its inability to identify *L. pneumophila* serogroups, which is essential for the epidemiological correlation of human cases and environmental colonization and (b) its inability to identify the most frequent species of *Legionella* in the environment, such as *L. anisa* and *L. longbeachae* (Mazzotta et al. 2021; Arrigo et al. 2022; Roussotte and Massy 2022). According to epidemiological reported cases of legionellosis, the number of *L. longbeachae* cases has increased markedly across Europe and parts of Asia (Whiley and Bentham 2011).

This means other potentially pathogenic *Legionella* species in water systems may go undetected, potentially leading to an underestimation of total *Legionella* risk. Therefore, even if MICA allows one to obtain a result in 48 h, the limited specificity means decisions might be based on incomplete knowledge of the total *Legionella* community present in the water, particularly when non‐*pneumophila* species are present. This information is essential for surveillance, outbreak investigations, and public‐health decision making.

In conclusion with further refinement of the MICA method, that is, testing longer incubation, it could be a very useful diagnostic tool, easily usable even in laboratories with little experience because it reduces the time to obtain confirmed results to 2 days, compared with the 10 days or more required by the plate‐culture method, it features very simple and rapid sample preparation and avoids the need for large‐volume filtration, colony isolation, and additional confirmation or identification. Moreover, the MICA test uses AI and automated imaging to detect and count microcolonies, reducing manual counting errors and human subjectivity in interpretation.

## Author Contributions

**Savina Ditommaso:** conceptualization, supervision, writing, review and editing. **Gabriele Memoli:** formal analysis. **Monica Giacomuzzi:** supervision, formal analysis, writing, review and editing. **Carla Streva:** formal analysis, review and editing (supporting). **Jacopo Garlasco:** data curation, writing, review and editing. **Carla M. Zotti:** resources. **Fabrizio Bert:** review (supporting).

## Ethics Statement

The authors have nothing to report.

## Conflicts of Interest

The authors declare no conflicts of interest.

## Data Availability

The original contributions presented in the study are included in the article; further inquiries can be directed to the corresponding author.
